# Programming Structural Symmetry and Emission in Low‐Dimensional Hybrid Perovskites via Time and Temperature

**DOI:** 10.1002/advs.76733

**Published:** 2026-07-23

**Authors:** Luiz G. Bonato, Sirous Khabbaz Abkenar, Balaji Dhanabalan, Quentin Evrard, Luca Goldoni, Sergio Marras, Giorgio Divitini, Milena P. Arciniegas

**Affiliations:** ^1^ Automated Nanomaterials Engineering Center for Convergent Technologies Istituto Italiano di Tecnologia Genova Italy; ^2^ Electron Spectroscopy and Nanoscopy Center for Convergent Technologies Istituto Italiano di Tecnologia Genova Italy; ^3^ Materials Characterization Facility Center for Convergent Technologies Istituto Italiano di Tecnologia Genova Italy

**Keywords:** Light emitters, Low‐dimensional perovskites, Structural reconfiguration

## Abstract

Layered hybrid perovskites offer exceptional opportunities for tuning optoelectronic properties through structural design. However, achieving reproducible and controllable functionality requires a detailed understanding of how their structures evolve during synthesis and processing. Here, we demonstrate programmable structural symmetry and emission control in benzylammonium‐based layered lead bromide perovskites through coupled time‐ and temperature‐dependent transformations. Under continuous shaking, the material evolves from orthorhombic platelets to a lower‐symmetry monoclinic phase composed of elongated, grooved microcrystals, accompanied by the emergence of broadband emission associated with exciton self‐trapping in the distorted monoclinic structure. By combining in situ and ex situ structural and optical characterization, we show that this transformation involves both lattice reorganization and redistribution of the organic component, consistent with a combination of distortion‐driven and solution‐mediated mechanisms. Thermal activation induces a reverse pathway toward a higher‐symmetry configuration resembling the original orthorhombic phase, accompanied by recovery of sharp excitonic emission. These results establish a direct link between processing history, metastable structural states, and emission behavior, providing a strategy for programming functional states in structurally soft layered perovskites toward adaptive and reconfigurable optoelectronic materials.

## Graphical abstract

We demonstrate programmable structural symmetry and emission in benzylammonium‐based low‐dimensional hybrid perovskites. Continuous shaking transforms orthorhombic platelets into a lower‐symmetry monoclinic phase with broadband emission, whereas thermal activation drives the reverse pathways toward a higher‐symmetry configuration resembling the original orthorhombic phase and restores sharp excitonic emission.

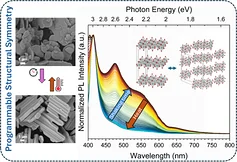

## 1 Introduction

Hybrid organic–inorganic metal halide perovskites are a versatile class of solution‐processable semiconductors with exceptional potential for optoelectronic applications [1]. In Pb‐based layered perovskites, the inorganic framework, composed of corner‐sharing [PbX_6_^4−^] octahedra, is confined by organic cations into two‐dimensional structures. This reduced dimensionality enables strong excitonic confinement [2], while the organic layers electronically decouple adjacent inorganic slabs and enhance environmental stability [3]. As a result, these materials exhibit highly tunable structure‐dependent optoelectronic properties, where subtle variations in lattice symmetry and octahedral connectivity can significantly modify their electronic structure and emission behavior. By tailoring their coordinating sites and steric volume [4], the [PbX_6_^4−^] octahedra connectivity can evolve from distorted corner‐sharing in two‐dimensional (2D) networks to edge‐ or face‐sharing motifs in lower‐dimensional (one or zero dimensional) configurations. Such connectivity changes strongly influence the electronic band structure, often broadening photoluminescence (PL) spectra through self‐trapped exciton formation [5].

Beyond compositional design, the intrinsic structural softness of layered perovskites introduces an additional degree of freedom for controlling their properties. Weak van der Waals interactions within the organic sublattice and the dynamic nature of organic cations render these systems highly responsive to external stimuli, enabling structural transformations such as interlayer slippage, distortions of the octahedral lattice, and symmetry changes [6, 7]. These transformations can be driven by variations in processing conditions, including time [8], temperature [9, 10], solvent environment [6], and external mechanical stimuli [11], and often lead to pronounced changes in optoelectronic properties, facilitating dynamic control over charge transport anisotropy [12], emission [13, 14], and switchable nonlinear optics [15]. Despite this tunability, achieving reproducible and controllable structural transformations remains challenging, in part because gradual structural evolution under dynamic conditions is difficult to capture and monitor experimentally.

Controlling access to metastable structural states provides a route toward programmable materials, where functionality can be tuned by navigating nonequilibrium energy landscapes [16, 17]. In layered perovskites, such metastable structures may emerge during growth or processing and can be stabilized through the interplay between kinetic and thermodynamic pathways. However, the coupling between time‐dependent structural evolution during synthesis and subsequent thermal relaxation remains unclear. In particular, how low‐symmetry, distortion‐accommodated phases form, evolve, and can be reconfigured through external stimuli has not been systematically investigated. Addressing this question is crucial for developing strategies to access and control functional metastable states in hybrid perovskites. In systems where multiple structural configurations are energetically accessible, the ability to navigate between them offers opportunities to tailor emission response, enable reversible color switching, and design adaptive materials. Moreover, understanding how such transformations reshape crystal morphology may enable the formation of anisotropic microstructures with distinct surface energies, offering additional opportunities for functionalization and dopant incorporation.

In this work, we address this gap by tracking bidirectional structural evolution in benzylammonium‐based layered perovskites as a function of both time and temperature. Using a room‐temperature injection synthesis, we identify a time‐dependent pathway in which increasing shaking time transforms orthorhombic, blue‐emitting platelets into monoclinic, elongated crystals with nanoscale grooves that exhibit broadband white‐light emission. Thermal annealing reveals a reciprocal temperature‐driven pathway that relaxes the distorted structure and restores narrowband blue emission. By combining ex situ and in situ structural probes with multimodal optical characterization, we establish a unified link between processing history, lattice symmetry, morphology, and emission behavior, demonstrating how structural states can be programmed through coupled temporal and thermal pathways.

## 2 Results and Discussion

### 2.1 Synthesis and Ex Situ Monitoring of Time‐Driven Structural Evolution

Motivated by our previous study on benzylammonium (BzA)‐based lead bromide layered perovskites, we focus here on BzA as the organic cation, which was shown to uniquely disrupt the conventional platelet morphology typically observed in Ruddlesden‐Popper layered perovskites when polar solvents are replaced with aromatic, nonpolar media. Under those conditions, the system forms elongated microcrystals with nanoscale surface grooves (‘grooved’ structures) that adopt a monoclinic phase composed of disconnected corner‐sharing octahedral ribbons and exhibit broadband white‐light emission [18]. This prior work established the existence of this unusual morphology and its associated optical response. However, how such highly distorted structures form has remained unclear. Here, we investigate how these structures emerge dynamically during synthesis and how their formation evolves under continuous exposure to the solvent environment.

The crystals are synthesized via a room‐temperature injection method in which PbBr_2_ is dissolved in a mixed aqueous‐toluene system, followed by rapid injection of benzylamine to initiate crystallization (Methods). The mixed aqueous‐toluene system was selected because the immiscibility between toluene and the HBr phase used to dissolve PbBr_2_ creates a diffusion barrier that slows the release of Pb species into the reaction medium. As previously reported for benzylammonium‐based layered perovskites, this reduced Pb availability alters the crystallization kinetics relative to fully miscible polar solvent systems, where PbBr_2_ is immediately accessible for octahedral formation [18]. The resulting nonequilibrium growth conditions promote alternative structural arrangements and facilitate access to the lower‐symmetry phase investigated in this work.

The structural evolution was monitored from the moment of injection up to 24 h of reaction time, both in static conditions and under continuous shaking, which maintains solvent‐mediated interactions, and drives time‐dependent structural evolution rather than passive aging. Immediately after benzylamine injection (defined as 0 h of reaction time), the product consists predominantly of small, platelet‐shaped microcrystals with lateral dimensions up to 7 µm (Figure 1a). This morphology is consistent with the rapid nucleation of a relatively symmetric layered phase (crystallographic structure shown in Figure 1b). In the absence of shaking, these platelets retain their morphology even after prolonged reaction time (Figure S1), indicating that the initial crystallization pathway favors the formation of a stable orthorhombic layered structure under static conditions. In contrast, when the reaction mixture is maintained under continuous shaking, a gradual time‐dependent transformation becomes apparent, indicating that the transformation is driven by solvent‐mediated restructuring under continuous mixing conditions rather than purely mechanical deformation. The extent of the transformation is also dependent on the shaking rate. Continuous shaking at 1700 rpm leads to complete conversion into the grooved monoclinic phase after 24 h, lower agitation rates result in incomplete transformation over the same reaction period, whereas static conditions do not induce the phase transition (Figure S2). These observations further support the role of the continuous mixing in promoting structural evolution.

**FIGURE 1:**
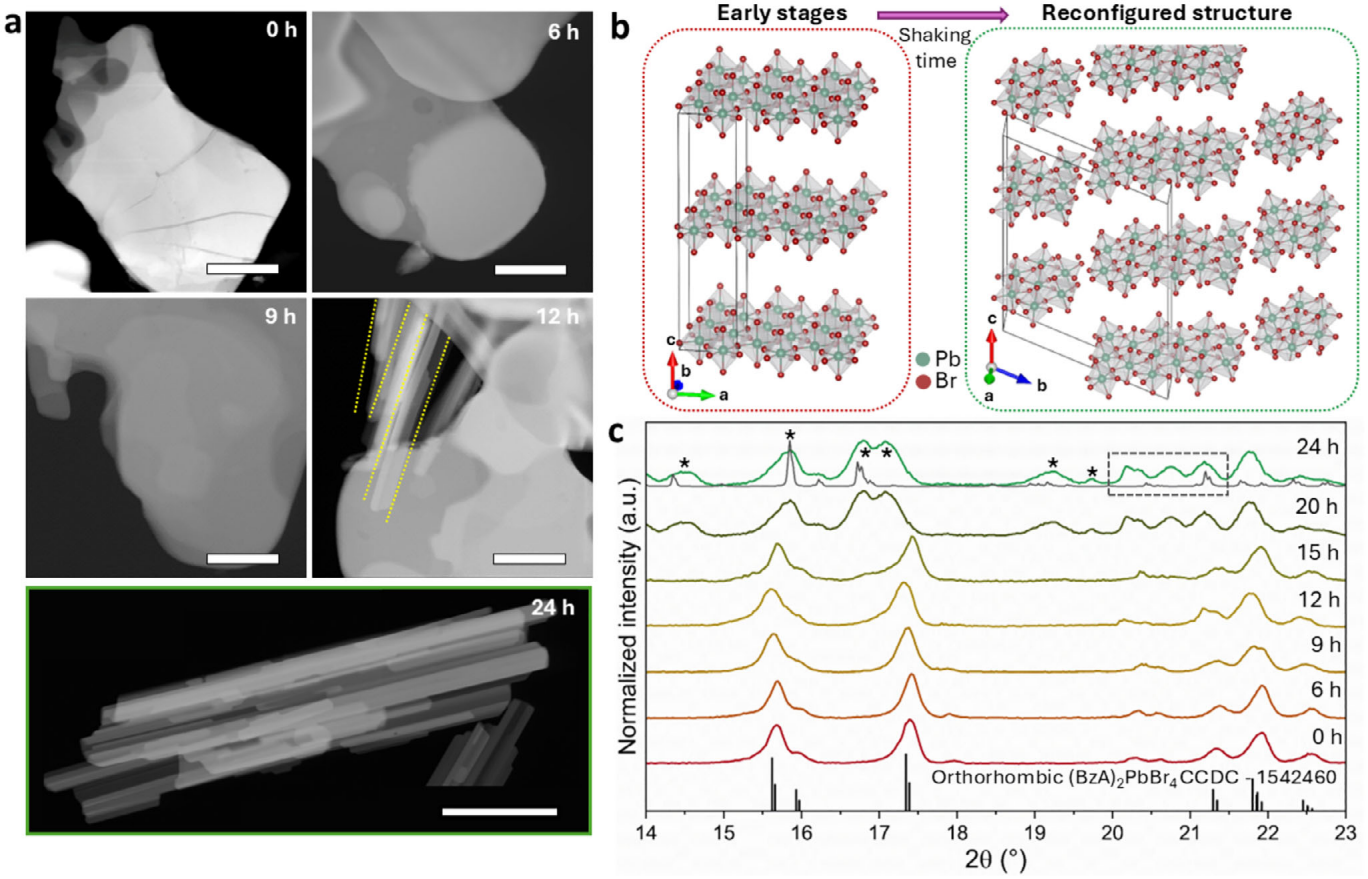
(a) STEM‐HAADF images of representative crystals collected at different reaction times (0, 6, 9, 12, and 24 h), illustrating the progressive transformation from platelets to elongated grooved microcrystals. At early times (0–9 h), the crystals maintain a platelet‐like morphology, while at intermediate times (∼12 h) anisotropic elongations begin to appear (yellow dashed lines). After prolonged shaking (24 h), the material consists predominantly of rod‐like crystals with pronounced longitudinal grooves. Scale bars: 2 µm (0, 6, 9, and 12 h) and 5 µm (24 h). (b) Structural models representing the orthorhombic phase observed at early reaction times (based on the reported crystal structure of orthorhombic (BzA)_2_PbBr_4_, CCDC 1542460) and the lower‐symmetry monoclinic phase identified previously by three‐dimensional electron diffraction [18], showing the reconfigured ribbon‐like inorganic layer. (c) Normalized XRD patterns of samples collected at different shaking times (0–24 h). The gradual emergence of additional reflections after ∼ 12 h indicates a structural transformation from the orthorhombic (BzA)_2_PbBr_4_ phase toward a lower‐symmetry monoclinic phase. Vertical lines correspond to the reference diffraction pattern of orthorhombic (BzA)_2_PbBr_4_ (CCDC 1542460), and the simulated diffraction pattern of the monoclinic phase determined by 3D electron diffraction [18] is shown below the 24 h sample for comparison. Asterisks mark reflections associated with the transformed phase, and the dashed box highlights the region where peak splitting and broadening become evident in the transformed structures.

Over the first several hours of synthesis (up to 9 h), the crystals largely preserve their platelet‐like morphology, suggesting that the early growth regime remains dominated by isotropic in‐plane crystal growth. X‐ray diffraction (XRD) patterns collected during this period remain unchanged and can be indexed to orthorhombic (BzA)_2_PbBr_4_ (space group Cmc2_1_) [19, 20], with only traces of benzylammonium‐bromide salt (Figure 1c). XRD patterns over an extended 2θ range are provided in Figure S3. Consistent with the diffraction data, Scanning Transmission Electron Microscopy—High Angle Annular Dark Field (STEM‐HAADF) images obtained at 6 and 9 h reveal equiaxed platelets with relatively uniform lateral dimensions and no evidence of directional elongation or faceting (Figure 1a). Such morphology indicates comparable in‐plane growth rates along the crystallographic axes, consistent with classical nucleation followed by diffusion‐mediated growth of the layered phase [21]. Together, these observations establish that the orthorhombic (BzA)_2_PbBr_4_ structure remains the dominant phase during the initial stage of time‐driven evolution.

After approximately 12–15 h of continuous shaking, the first signatures of structural reconfiguration become apparent. The XRD pattern begins to exhibit additional weak reflections, most notably a shoulder near 2θ ≈ 17°, which cannot be indexed to the orthorhombic lattice. The evolution of the 15–7° region reflects overlapping contributions from the orthorhombic phase and the emerging lower‐symmetry phase, rather than a simple peak shift. By 15 h, these features become more pronounced, while the characteristic orthorhombic reflections remain clearly visible. The simultaneous presence of both sets of reflections indicates the formation of a heterogeneous mixture of two solid phases, in which a portion of the material preserves the original (BzA)_2_PbBr_4_ structure while another fraction begins to transform into a new crystalline phase. This structural coexistence is mirrored by the corresponding STEM‐HAADF images (Figure 1a).

At these intermediate reaction times (12–15 h), platelet‐like crystals characteristic of the initial layered phase are still observed, but they coexist with newly formed elongated microcrystals with high aspect ratios (in the 5–10 range), Figure 1a, 12 h highlighted with dotted yellow lines. The appearance of these anisotropic structures marks the onset of the morphological transformation that ultimately leads to the grooved monoclinic phase (arrangement of the inorganic layers displayed in Figure 1b). The gradual evolution of both diffraction signatures and crystal morphology over this time interval suggests a progressive, time‐driven restructuring process rather than an abrupt single‐step transition, denoting that the system can be driven between distinct structural states through controlled processing pathways.

Upon further shaking, the structural transformation progresses until it becomes essentially complete: after 20 h, the XRD patterns are dominated by a new set of broadened diffraction peaks, while the reflections associated with the orthorhombic phase become strongly attenuated or disappear altogether (Figure 1c). Several new features emerge at 2θ around 14.5°, 15.8°, 17,1°, 19.2°, and 21.2°, indicating substantial reorganization of the crystal lattice and formation of a new crystalline phase. Comparison with the XRD pattern of the BzA‐Br salt (Figure S4) confirms that these features originate from the transformed perovskite structure rather than residual precursors. The appearance of additional reflections is consistent with a reduction in crystallographic symmetry, since symmetry lowering relaxes systematic absences and increases the number of Bragg reflections allowed.

This interpretation agrees with our previous structural analysis based on three‐dimensional electron diffraction and Rietveld refinement [18], which showed that the fully transformed material adopts a C‐centered monoclinic structure, which indicates a pronounced symmetry reduction relative to the orthorhombic phase and reflects significant lattice distortion within the inorganic framework. The corresponding structural model from [18] was used to generate the simulated diffraction pattern shown in Figure 1c and Figures S3 and S4.

To further probe the role of the organic component during the transformation, we performed quantitative ^1^H NMR on dissolved samples collected at 0 and 24 h (Figure S5). For quantitative comparison, identical masses of the 0 and 24 h samples (30 mg) were dissolved in the same volume of deuterated dimethylsulfoxide (DMSO‐d6, 600 µL) prior to analysis. The total benzylammonium concentration (BzA^+^) present in the dissolved ensemble of crystals was determined by comparing integrated aromatic proton signals to an external standard solution of dimethylsulfone (Methods). Because solution‐state ^1^H NMR is performed after complete dissolution of the samples, the measured BzA^+^ concentration represents an average value of the dissolved crystals and does not provide direct information on the organic cation content of individual crystals. The 0 h sample exhibits a higher BzA^+^ concentration (ca. 165 mm), whereas the 24 h sample shows a reduced value (ca. 148 mm), indicating a lower relative content of the organic cation in the transformed structures needed to stabilize the ribbon‐like inorganic framework.

The morphological evolution observed by STEM‐HAADF follows the same trend. At 24 h, the crystals consist almost exclusively of elongated, rod‐like microcrystals with sharply faceted edges and pronounced longitudinal grooves (Figure 1a, 24 h). These highly anisotropic crystals represent the fully transformed state and are associated with stabilization of the lower‐symmetry monoclinic phase coupled to the lowered‐symmetry lattice. Further shaking does not induce additional morphological changes, and the elongated grooved structures remain stable even after prolonged shaking up to 72 h (Figure S6). The pronounced peak broadening observed in the XRD patterns at this stage is consistent with the increased structural complexity of the transformed structure. However, because the diffraction patterns contain overlapping contributions from coexisting phases, a quantitative assessment of lattice strain is not reliable. The broadening is therefore attributed qualitatively to the progressive structural reorganization accompanying formation of the lower‐symmetry phase. Together, the combined diffraction and microscopy results reveal a progressive time‐dependent pathway in which the initial orthorhombic platelet phase evolves into a strained monoclinic phase characterized by elongated grooved crystals. These observations indicate that the structural transformation develops through a progressive reorganization pathway, which is further reflected in the evolution of crystal morphology and optical response.

To verify that the observed structural reconfiguration is not accompanied by major compositional changes, we performed spatially resolved STEM‐EDX (Energy‐Dispersive X‐ray spectroscopy) analysis on representative crystals collected at different reaction times. Elemental maps of Pb and Br reveal a homogeneous distribution across both platelets and grooved morphologies (Figure S7), with no evidence of compositional segregation or impurity phases. Although local variations in the measured Pb ratios are observed between individual regions of interest, these fluctuations are attributed primarily to differences in crystal thickness, orientation, and detector geometry, all of which can influence quantitative STEM‐EDX measurements of relatively large microcrystals. Importantly, the analysis does not reveal any systematic compositional trend associated or elemental segregation associated with the structural transformation (Table S1). These results support the conclusion that the time‐driven evolution from orthorhombic platelets to monoclinic grooved crystals arises from structural reorganization rather than compositional modification.

### 2.2 Transformation Mechanism and Impact on the Emission Properties

The observed structural evolution originates from progressive distortion of the layered framework, which destabilizes the initial high‐symmetry configuration and drives the transformation toward a lower‐symmetry structure. At early stages of synthesis, the orthorhombic lattice stabilizes broad, low‐energy facets and favors 2D platelet growth [22]. In this configuration, the layered structure remains relatively strain‐free and supports isotropic lateral expansion of the inorganic layers.

Under continuous shaking, however, the soft organic–inorganic interfaces begin to experience shear and interlayer slippage: the relatively weak van der Waals interactions between organic cation layers facilitate local lattice distortions and partial displacement of adjacent layers, as previously reported in layered perovskites such as (BA)_2_PbI_4_ and (PEA)_2_PbI_4_ [23, 24]. As mechanical energy accumulates over time, these distortions progressively destabilize the high‐symmetry orthorhombic configuration, promoting lattice deformation and local strain accumulation. Such strain effects are well known in halide perovskites to arise from lattice mismatch, structural softness, and nonequilibrium growth conditions, and can significantly influence structural stability and optoelectronic response [25, 26, 27, 28]. Recent studies have further emphasized that strain can influence structural anisotropy, octahedral distortions, phase stability, and the resulting optical and electronic properties of hybrid perovskites, highlighting the strong coupling between lattice evolution and functional response [29, 30]. A recent review has also emphasized the central role of both static and dynamic lattice distortions in the optical and electronic response of low‐dimensional hybrid perovskites [7].

As the lattice evolves toward a lower‐symmetry configuration, isotropic in‐plane growth becomes unfavorable. As a consequence of this structural instability, crystal growth becomes anisotropic by redirecting growth along the less sterically constrained directions (Figure 2a,b) to minimize its elastic energy. Similar symmetry‐breaking growth mechanisms have been reported in layered perovskites, where directional intermolecular interactions and lattice anisotropy drive preferential crystal growth along specific crystallographic directions [12, 21, 31]. In the present system, the emergence of elongated microcrystals suggests that the balance of intermolecular interactions influencing crystal growth changes during the transformation. Due to the aromatic nature of the organic cation, modifications in noncovalent interactions such as C─H···*π* and *π*–*π* contacts may contribute to the development of growth anisotropy. Similar relationships between organic‐cation interactions, lattice distortions, and structural response have been reported in hybrid perovskites, where hydrogen‐bonding interactions affect octahedral distortions and thus electronic properties [32]. Although the present data do not directly resolve these molecular changes, their influence is consistent with the observed symmetry breaking and preferential crystal growth along the *c*‐axis, yielding elongated microcrystals.

**FIGURE 2:**
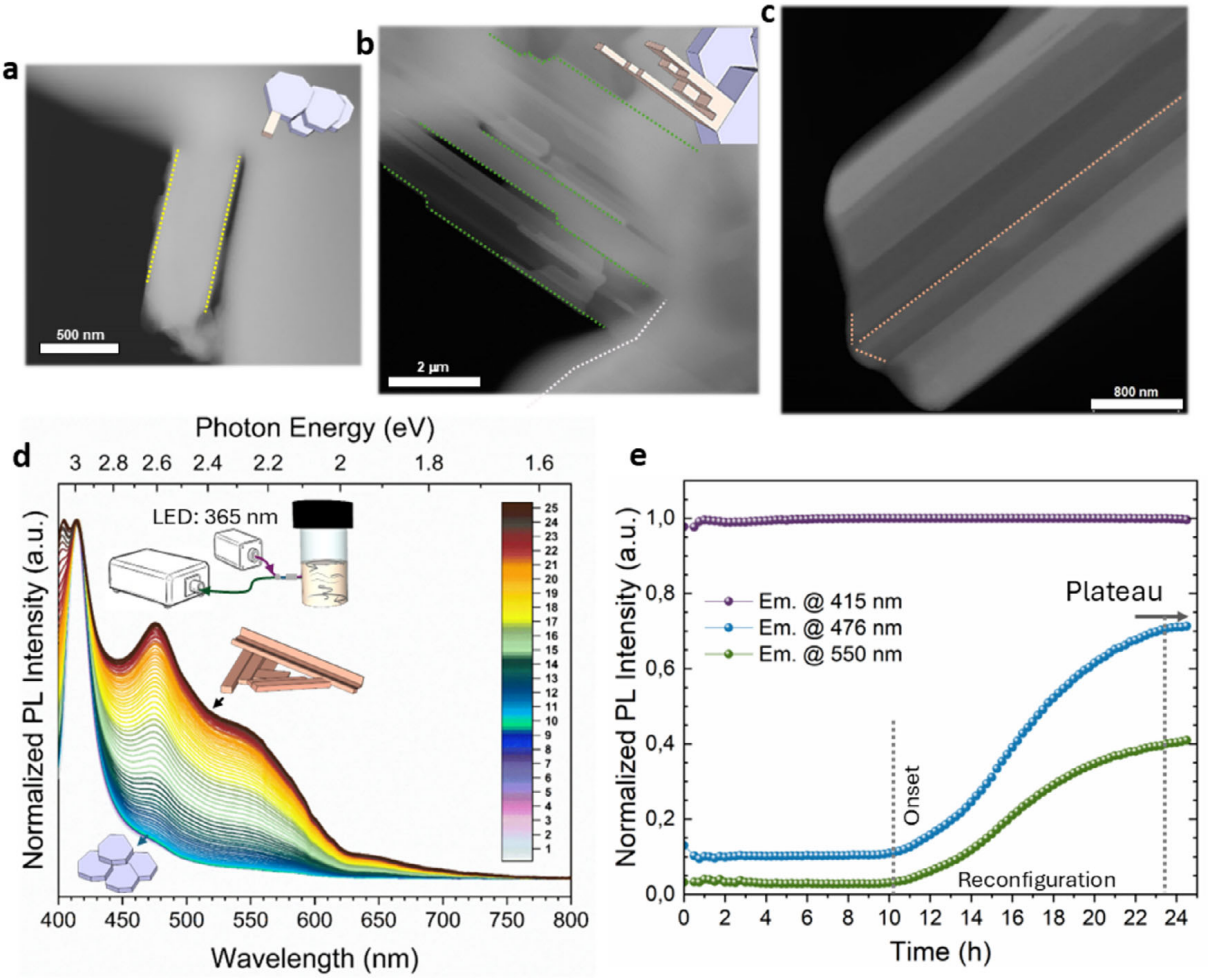
(a–c) STEM‐HAADF images highlighting the anisotropic growth and groove formation in elongated microcrystals at advanced reaction stages: (a) early elongation showing preferential growth direction (yellow dashed lines); (b) Intermediate stage illustrating facet development and anisotropic extension (green dashed lines); (c) Fully developed grooved microcrystal, where longitudinal grooves define the segmented morphology (orange dashed lines). (d) In situ PL collected during continuous shaking (0–24 h), showing the evolution from narrowband‐edge blue emission (ca. 415 nm) to broadband emission extending across the visible range. The color scale represents reaction time. (e) Normalized PL intensity at selected wavelengths (415, 476, and 550 nm) as a function of reaction time, highlighting the onset (around 10–12 h) and progressive development of low‐energy emission channels associated with the structural reconfiguration. The plateau at longer times indicates completion of the transformation into the broadband‐emitting phase.

As the transformation progresses, the accumulated mismatch between reorganized (ribbon‐like) octahedra segments generates local lattice distortions within the crystals. To further relieve elastic energy, elongated crystals grow and develop longitudinal groove‐like indentations along specific crystallographic directions (Figure 2c). These grooves represent controlled partial faceting of the crystal surface, analogous to re‐entrant grooves observed in strained metal crystals, where internal lattice mismatch is accommodated through surface reconstruction rather than fracture [33, 34, 35]. In the present case, groove formation acts as an additional strain‐relief mechanism, stabilizing the elongated monoclinic crystals despite the substantial lattice distortion introduced during the time‐driven structural transformation. The resulting monoclinic configuration appears to accommodate the accumulated distortions more effectively than the original orthorhombic phase through its ribbon‐like inorganic framework and the formation of longitudinal grooves, allowing the transformed structure to remain under continued shaking.

Since the optical response of layered hybrid perovskites is highly sensitive to crystallographic structure and thus microstructural organization, we next investigated how the time‐driven structural transformation described above influences the emissive behavior of the system. Continuous in situ PL measurements were therefore performed during the synthesis under constant agitation (Figure 2c), enabling real‐time monitoring of the optical evolution as the structural reconfiguration progresses. At early reaction times (t ≤ 9 h), when the sample predominantly consists of orthorhombic platelet‐like crystals, their PL spectra (Figure 2d) display a sharp deep‐blue emission peak centered at ∼415 nm (2.99 eV) with a full width at half‐maximum (FWHM) of approximately 24 nm (173 meV).

This narrow emission is characteristic of free‐excitonic recombination in 2D RP layered perovskites and originates from radiative recombination within the Pb─Br inorganic framework [5, 36, 37]. The gradual increase of the higher‐energy shoulder near 405 nm with shaking time may reflect changes in the relative contribution of excitonic emission as the crystal morphology evolves from platelets to elongated grooved microcrystals. In layered perovskites, excitonic transitions are sensitive to structural organization, crystal orientation, and dipole alignment [2, 12, 38], all of which can influence the relative intensity of the different excitonic components [12, 39]. As the structural transformation begins (12–15 h), coinciding with the emergence of elongated morphologies and phase coexistence observed in XRD and STEM analysis, the PL spectra gradually broaden and develop a low‐energy tail extending across the 500–750 nm range.

This evolution is clearly visible in the PL profile as a function of time (Figure 2d) and is further illustrated by the corresponding PL intensity contour map (Figure S8). Quantitative analysis using the emission intensity at 415 nm as a reference, it shows that the relative intensity of the low‐energy emission increases following a sigmoidal trend with reaction time, reaching saturation at around 24 h. This sigmoidal evolution, together with the coexistence of distinct structural phases observed by XRD, suggests that the transformation proceeds through a gradual reorganization process rather than a purely continuous deformation of the initial lattice. In addition to strain‐driven structural rearrangement, a complementary mechanism involving partial dissolution and recrystallization at the crystal surface cannot be excluded [6, 8, 40]. In such a scenario, the aqueous phase (from HBr used for dissolving PbBr_2_ powders) may locally dissolve the edges or defect sites of the orthorhombic layered structures, enabling the nucleation and growth of the lower‐symmetry monoclinic phase. Distinguishing between these possible contributions and establishing their relative importance will require further investigation of the coupled solvent‐mediated and lattice‐relaxation processes involved in the transformation. The present results establish the overall transformation pathway and identify its structural and optical signatures. More broadly, they indicate that the structural evolution is dictated by the combined influence of the solvent environment, shaking conditions, reaction time, and the intrinsic structural adaptability of the BzA layer, which together promote the strain‐driven reconfiguration of the layered lattice. A detailed theoretical description of how these factors interact, including solvent‐mediated mass transport, local strain accumulation, and lattice relaxation, therefore remains an important direction for future investigation.

In addition to the development of broadband emission, a weak feature centered at 476 nm emerges at prolonged reaction times. The appearance of this component is unlikely to originate from excitonic transitions associated with phases containing a larger number of inorganic layers, as such structures are stabilized by smaller organic cations and are not supported by the structural characterization of the present system. This feature is more likely associated with intermediate localized excitonic states arising from the structural heterogeneity that develops during the transformation. The coexistence of orthorhombic and monoclinic domains can generate locally distorted environments, where lattice relaxation, variations in octahedral connectivity, and edge‐related distortions produce emissive states with energies intermediate between the band‐edge excitonic emission and the strongly localized self‐trapped excitons [41, 42]. The progressive increase of the 476 nm component therefore likely reflects the increasing population of such locally distorted environments as the structural transformation proceeds.

The most relevant optical change, however, is the emergence of a strong broadband emission at longer reaction times. When the transformation is complete (around 24 h), the PL spectrum is dominated by a broad emission band, characteristic of self‐trapped exciton recombination in distorted low‐dimensional perovskites [5, 43, 44]. In such systems, enhanced electron‐phonon coupling, typically accompanied by local lattice distortion, can dynamically induce structural relaxation that transiently traps photoexcited carriers, creating localized emissive states below the band edge.

To provide a structural metric for the transformation, Table 1 compares representative Pb─Br─Pb bond angles extracted from the available crystallographic models of the orthorhombic and monoclinic phases. Although these values are not derived from newly refined crystal structures of the samples, they provide a useful measure of the relative increase in lattice distortion associated with the structural reconfiguration. The comparison reveals substantially larger deviations from ideal octahedral connectivity in the monoclinic phase, with enhanced lattice distortion at ribbon edges (Table 1 and Figure 3), providing a favorable environment for exciton self‐trapping. Additional evidence supporting the self‐trapped exciton assignment has been reported for the same monoclinic phase in our previous work [18], including power‐dependent PL measurements showing different excitation‐density scaling for excitonic and broadband emission, the absence of saturation of the broadband component over the investigated excitation range, comparable time‐resolved PL decay profiles across the monitored emission wavelength (Figure S12), and the persistence of broadband emission after defect passivation treatment. These observations support an intrinsic self‐trapping mechanism rather than defect‐mediated PL [18, 45, 46].

**TABLE 1:** Representative in‐plane Pb─Br─Pb bond angle extracted from the reported orthorhombic crystal structure of the (BzA)_2_PbBr_4_ (CCDC 1542460), whose diffraction pattern matches that of the 0 h sample, and from the inorganic framework of the monoclinic phase of the grooved structures (t = 24 h) determined previously by 3D electron diffraction [18]. The values are presented to illustrate the relative increase in lattice distortion associated with the structural reconfiguration. The corresponding partial structural model of the monoclinic phase is provided in the Supporting Information. Crystallographic views are shown in Figure S9.

Crystal structure/Morphology	Symmetry	Segment	In‐plane bond angle (°)
Orthorhombic/Platelet	Cmc2_1_	Pb1‐Br2‐Pb1	149.94
		Pb1‐Br3‐Pb1	149.92
Monoclinic/Grooves	C2	Pb1‐Br1‐Pb1	128.0
		Pb1‐Br4‐Pb2	129.4
		Pb1‐Br5‐Pb2	147.3
		Pb2‐Br9‐Pb3	150.1
		Pb2‐Br8‐Pb3	142.8

**FIGURE 3:**
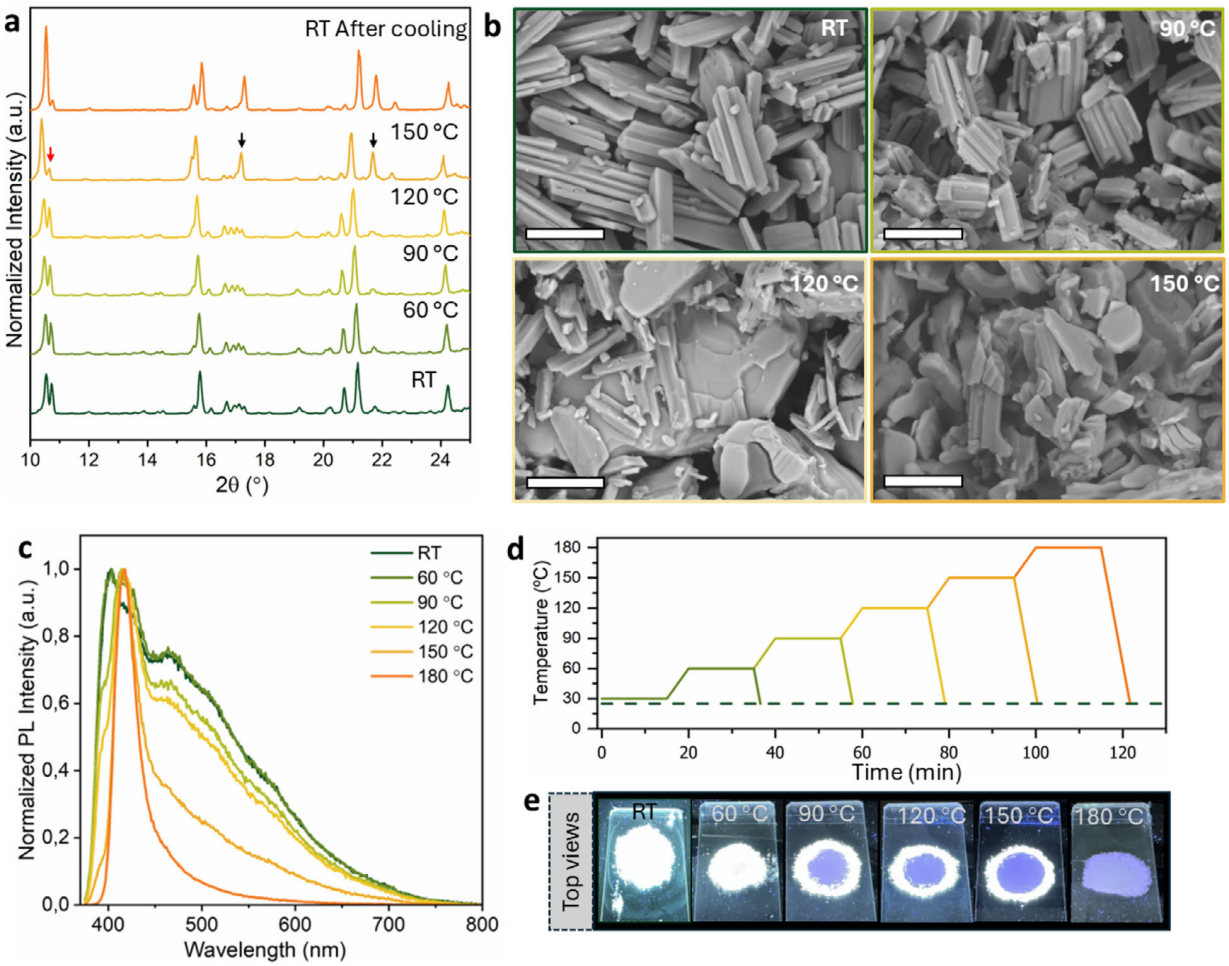
(a) In situ heating XRD patterns collected from the groove crystals at room temperature (RT), 60°C, 90°C, 120°C, and 150°C (with 15 min dwell time at each temperature), followed by cooling back to 25°C, showing progressive structural reconfiguration to the orthorhombic phase, which is preserved upon cooling. (b) Ex situ SEM images illustrating the temperature‐dependent morphological evolution. The elongated, grooved microcrystals observed at RT are preserved up to 90°C, while partial relaxation is observed at 120°C, followed by collapse of the grooved morphology and formation of smoother, compact crystals at 150°C. Scale bars: 5 µm. (c) Normalized PL spectra of the samples after thermal treatment at the temperature indicated in the legend for each line, showing the suppression of broadband emission and recovery of narrow excitonic emission with increasing temperature. (d) Temperature profile used for the stepwise thermal treatment, indicating dwell temperatures and heating sequence. (e) Photographs under UV illumination show the corresponding color evolution from broadband white emission (RT) to dominant blue emission at higher temperatures.

These results show that the reconfiguration from orthorhombic platelets to strained monoclinic grooved structures progressively increases lattice distortion and structural heterogeneity, thereby activating self‐trapped exciton pathways, adding a broadband white emission channel.

### 2.3 Thermal Relaxation and Optical Response of the Mechanically Stabilized Monoclinic Phase

The time‐driven structural transformation described above leads to the formation of a strain‐accommodated monoclinic phase characterized by elongated, grooved microcrystals and broadband emission. An important question is whether this distorted structure is thermodynamically stable or can be relaxed through thermal activation. To address this, we investigate the thermal response of the 24 h transformed materials, focusing first on the structural and morphological evolution under controlled heating conditions. This approach allows us to determine whether the time‐induced symmetry‐lowering of the structure can be reversed or reconfigured through temperature‐driven lattice relaxation.

We first assessed the thermal stability of the material using thermogravimetric analysis (TGA) to define the temperature range over which structural transformations can be studied without chemical decomposition. As shown in Figure S10a, the material remains stable up to ∼250°C, confirming that the structural changes observed at lower temperatures arise from lattice reconfiguration rather than degradation. Above this temperature, a major mass‐loss event (52.3%) occurs, corresponding to the decomposition of the organic layer through dissociation of the benzylammonium, leaving PbBr_2_ as the primary inorganic residue [47]. Having established the relevant temperature window, we investigated thermal behavior using differential scanning calorimetry (DSC, Methods). During the first heating cycle (Figure S10b), a distinct endothermic event appears with an onset near ∼ 90°C. Since no mass loss occurs below ∼250°C, this feature is assigned to a thermally induced structural transition rather than decomposition. The DSC signal indicates that a thermally activated process begins near 90°C, whereas significant changes in the diffraction pattern become apparent only at higher temperatures. This suggests that the early stages of the transformation involve structural rearrangements that precede the development of detectable long‐range crystallographic reorganization. Previous studies have shown that thermally induced strain can drive progressive structural evolution in hybrid perovskites, with structural and optoelectronic changes developing over a temperature range. No corresponding exothermic feature is observed upon cooling, and subsequent heating‐cooling cycles show no additional thermal events, indicating that the transformation is irreversible. These results indicate that a thermally activated relaxation process begins near 90°C and progressively drives the lattice reorganization observed at higher temperatures.

To directly probe the structural evolution associated with this transition, we performed in situ temperature‐dependent XRD using a stepwise heating protocol (Methods). Figure 3a displays a collection of diffraction patterns depending on the temperature for a 2θ region in which major changes were observed: initially, they remain essentially unchanged from room temperature up to 120°C, indicating that the grooved monoclinic phase is stable within this temperature range. A pronounced transformation emerges at 150°C: the low‐angle reflection near 2θ ≈ 10.8° is strongly attenuated (red arrow), the reflections in the 15–17.5° region reorganize into a dominant feature at ∼17.3° (black arrow), and the feature near ∼21.8° becomes significantly enhanced (black arrow). These changes indicate a temperature‐driven reconfiguration of the monoclinic lattice. Importantly, after cooling back to room temperature, the diffraction pattern closely resembles that of the high‐temperature state, confirming that the transformation is not reversible. Note that Figure 3a displays the 2θ region in which major changes were observed.

The corresponding microstructural evolution is revealed by ex situ SEM images (Figure 3b). At room temperature, the material consists of elongated, grooved microcrystals with sharp facets.

This morphology is preserved up to 90°C. At 120°C, the first signs of relaxation appear: facets become rounded, and groove depth decreases, indicating partial reconstruction of the strain‐accommodating structure. At 150°C, the grooved architecture collapses, yielding smoother and more compact crystals. At 180°C, grooves are fully eliminated, and the population of elongated microcrystals is significantly reduced. These observations demonstrate that the grooved monoclinic morphology is metastable at relatively high temperatures, progressively destabilized by thermal activation.

The optical response directly reflects this structural relaxation. Ex situ PL spectra show that increasing annealing temperature progressively suppresses the broadband emission characteristic of the grooved monoclinic phase, ultimately leaving a spectrum dominated by a narrow excitonic emission centered near ∼420 nm (Figure 3c). Samples heated to 60°C–120°C retain a residual broadband tail, consistent with partial persistence of groove‐bearing domains. In contrast, at ≥150°C, the broadband component nearly disappears, yielding a sharp band‐edge emission similar to that of the pristine orthorhombic platelets. This behavior represents the inverse optical pathway of the time‐driven transformation: whereas prolonged agitation induces lattice distortions and activates broadband emission, thermal treatment relaxes these distortions and restores delocalized excitonic recombination. The observed strong coupling between lattice strain, structural reorganization, and optical response is consistent with recent studies showing that thermally induced strain evolution can substantially modify the structural and optoelectronic properties of hybrid perovskites [48]. Figure 3d,e shows the thermal sequence applied to the samples and the evolution of the emission color under UV illumination. Comparison of the XRD pattern of the thermally treated sample (150°C, cooled to room temperature) with that of the orthorhombic (BzA)_2_PbBr_4_ reference (Figure S11) reveals that the principal diffraction features can be indexed within the same structural framework, indicating that the thermal treatment drives the system toward a higher‐symmetry configuration. Both the in situ and ex situ XRD measurements show a systematic shift of the reflections toward lower 2θ values, corresponding to an approximately 0.5% expansion of the lattice parameter, together with enhanced preferred orientation along the 00l direction. Although these observations indicate that the thermally relaxed phase closely resembles the original orthorhombic lattice, the diffraction patterns do not fully coincide and a complete structural refinement of the relaxed phase is not currently available. We therefore describe it as a higher‐symmetry configuration that is structurally similar to, but not identical with, the pristine orthorhombic phase. This interpretation is further supported by the irreversible DSC signature (Figure S10b).

Direct evidence of this relaxation process is provided by in situ heating during STEM‐HAADF imaging. The full temperature profile applied during the in situ heating experiment is summarized in Figure S13. At room temperature, elongated grooved microcrystals exhibit well‐defined grooves and sharp facets. (Figure 4a).

**FIGURE 4:**
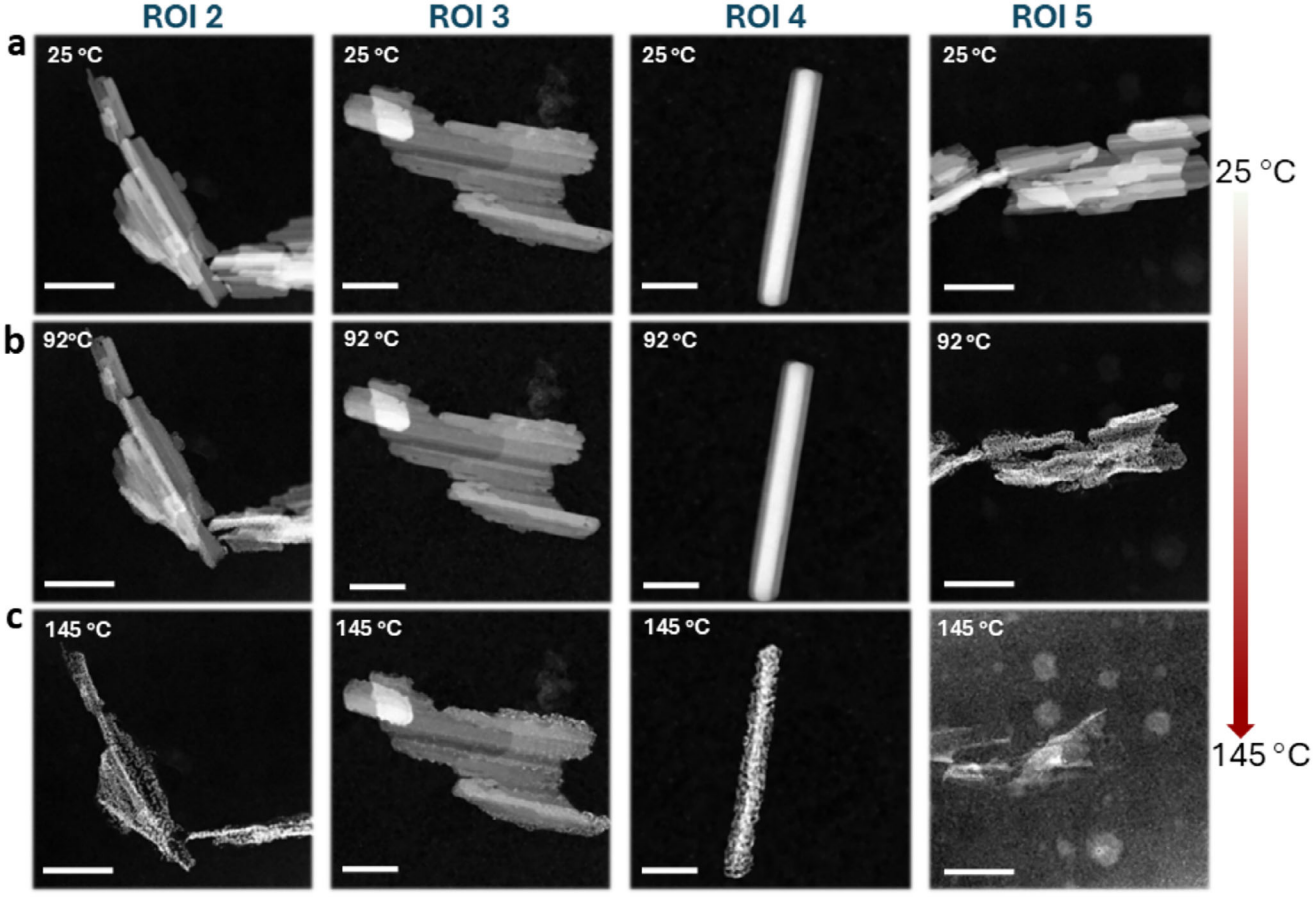
STEM‐HAADF images of representative ROIs during the in situ heating experiments at (a) 25°C, (b) ∼92°C, and (c) ∼145°C, showing the progressive degradation of elongated grooved microcrystals from well‐defined structures to collapsed morphologies. Scale bars: 5 µm (ROI 2 and 5), 2 µm (ROI 3), and 1 µm (ROI 4). ROI 1 was used as a control, and it is shown in Movie S1. The images correspond to representative snapshots acquired during continuous heating at 10°C min^−1^, with an intermediate isothermal hold of approximately 7 min at ∼92°C.

The temperature was initially ramped from room temperature to 200°C at a rate of 10°C min^−^^1^. A region labeled as ROI 1 was continuously exposed to the electron beam through the experiment and is therefore reported only as a quantitative control (Movie S1). To minimize beam‐induced effects, the remaining regions (ROI 2–ROI 5) were imaged for a few frames at selected temperatures. The temperature ramp was paused at ∼92°C and maintained for approximately 7 min, during which additional ROIs were examined under identical thermal conditions (Figure 4b). Heating was then resumed at 10°C min^−^^1^ from 92°C. A rapid morphological breakdown occurs near 145°C, with the collapse of the grooved architecture taking place over the course of minutes (Figure 4c and Movie S2). This timescale is consistent with the ex situ thermal annealing experiments, where comparable morphological changes are observed after 15 min dwell times between 120°C and 150°C.

Upon heating, groove softening begins near ∼75°C, followed by progressive collapse of the grooved architecture. The temperature ramp was subsequently paused at ∼92°C, and the sample was held isothermally for ∼7 min to assess the response of additional ROIs under identical thermal conditions (Figure 4b). Heating was then resumed at 10°C min^−^^1^ from 92°C. A rapid morphological breakdown occurs near 145°C where crystals lose structural integrity (Movie S20). These observations confirm that the distortion‐stabilized monoclinic phase undergoes irreversible structural relaxation. Note that these observations were obtained from individual grooved structures heated under high vacuum and exposed to an electron beam intermittently. Consequently, additional degradation pathways may be activated and the exact onset temperatures may differ from those observed under ex situ heating conditions. Nevertheless, the structural relaxation observed during the in situ STEM experiments is fully consistent with the separated temperature‐dependent XRD, SEM, and PL measurements performed without electron beam irradiation (Figure 3), confirming that the transformation is intrinsically driven by heating.

## 3 Conclusions

In summary, this work establishes a direct relationship between structural evolution pathways and optoelectronic functionality in layered perovskites. We demonstrate that a combined solvent‐mechanical forces‐mediated evolution during synthesis drives a progressive transformation from a high‐symmetry orthorhombic phase platelets to a lower‐symmetry monoclinic configuration characterized by anisotropic, grooved microstructures (formed as stress releasers), and broadband emission. This transformation reflects a coupled process involving lattice distortion, anisotropic crystal growth, and structural reorganization. Thermal activation induces relaxation of the distorted lattice, leading to the collapse of the grooved morphology and recovery of a higher‐symmetry configuration of the inorganic layer with dominant excitonic emission. These results reveal that the system can be driven between different structural states through controlled processing pathways, highlighting the role of nonequilibrium dynamics in defining accessible material configurations. They also show how structural reconfigurations in soft hybrid semiconductors can be harnessed as a design parameter to tune symmetry, morphology, and emission properties. Although demonstrated here in benzylammonium‐based systems, this behavior may be relevant to other structurally soft layered perovskites. However, the extent to which a similar transformation pathway can be activated is anticipated to be dependent strongly on the nature of the organic cation, as lattice flexibility, intermolecular interactions, and structural adaptability are highly organic cation dependent. Future efforts will be directed toward understanding how these factors determine the access to metastable structural states across broader families of layered perovskites, enabling controlled phase switching, strain‐responsive photonic behavior, and tunable broadband emission.

## 4 Methods

### 4.1 Materials

Lead(II) bromide (PbBr_2_, 98%), hydrobromic acid (48wt.%, aqueous solution), benzylamine (BzA, 99%), and toluene (99.7%) were obtained from Sigma–Aldrich and used as received, without further purification.

### 4.2 Synthesis of BzA‐Based Layered Perovskites

In a 4 mL glass vial, PbBr_2_ (110 mg, 0.30 mmol) was completely dissolved in 48 wt.% HBr (140 µL, 1.2 mmol), followed by the addition of 2 mL of toluene. The mixture was shaken vigorously in an orbital shaker at 1700 rpm for 5 min. Next, benzylamine (126 µL, 1.154 mmol) was rapidly injected into the precursor solution and immediately mixed using a vortex mixer for 1 min. The reaction vial was then left under continuous orbital shaking at 1700 rpm for the selected times, from 0 (pristine, unshaken), 1, 6, 9, 12, 15, 20, 24 h, and 3 days. The resulting crystals were collected by centrifugation at 5000 rpm for 5 min, redispersed in 2 mL of toluene. This washing/redispersion step was repeated three times to remove unreacted species. The crystals were finally redispersed in toluene, transferred onto filter paper in a Büchner funnel, washed several times with toluene and hexane, and dried under vacuum. All syntheses were performed under ambient laboratory conditions. No measurable temperature increase of the reaction was observed during shaking. Given the low reaction volume and efficient heat exchange with the environment, any temperature rise is estimated to be negligible compared with the temperatures required to induce the thermally driven transformation, that is, above 90°C.

### 4.3 Thermal Treatment of the BzA‐Grooved Structures

50 mg of the crystals prepared using 24 h of shaking (white‐emitting under UV light) was spread between two glass slides, gently pressed to form a uniform layer, and secured using adhesive tape on a temperature‐controlled hotplate. The sample was subjected to stepwise thermal annealing following a controlled heating profile. Starting from room temperature, the hotplate temperature was increased to 60°C, 90°C, 120°C, 150°C, and 180°C at a ramp rate of 5°C min^−^^1^ and held for 15 min at each target temperature. At the end of each dwell period, a small portion of the material was extracted for further analysis.

### 4.4 Structural Characterization

Ex situ XRD measurements were performed on a Malvern PANalytical Empyrean diffractometer equipped with a Cu Kα radiation source (λ = 1.5406 Å), operating at 45 kV and 40 mA, and configured in parallel‐beam geometry with a PIXcel3D 2 × 2 area detector (28.4 × 28.4 mm, 516 × 516 pixels). Diffraction patterns were collected over the 2θ range of 5°–42° using a step increment of 0.02°, dwelling 1 s per step over the scan range. All measurements were carried out on finely ground crystals deposited on zero‐background silicon substrates to minimize substrate contributions. XRD analysis was performed on the crystals obtained after the different shaking times for direct comparison of structural evolution as a function of shaking time.

In situ XRD measurements were collected using a third‐generation Empyrean diffractometer (Malvern‐PANalytical, Westborough, MA) equipped with a 1.8 kW Cu Kα X‐ray tube operating at 45 kV and 40 mA, automated prefix iCore‐dCore optical modules for the incident and diffracted beam paths, and a PIXcel3D area detector operating in 1D mode. Temperature‐dependent XRD measurements, from 30°C to 180°C, were enabled by an Anton Paar TTK600 temperature‐controlled chamber operated under ambient atmosphere. The powders were placed on a zero‐background silicon substrate to minimize substrate contributions.

For scanning transmission electron microscopy, BzA‐based lead bromide microcrystals were dispersed in toluene, gently sonicated, and drop‐cast onto carbon‐film‐coated Cu TEM grids. Scanning Transmission Electron Microscopy (STEM)‐High‐Angle Annular Dark‐Field (HAADF) imaging was performed using an image‐Cs‐corrected JEOL JEM‐2200FS microscope equipped with a Schottky field‐emission gun, operated at 200 kV. Compositional analysis and elemental distribution maps were acquired using a Bruker XFlash 5060 silicon‐drift detector (SDD) for energy‐dispersive X‐ray (EDX) spectroscopy. The EDX analysis was used primarily to identify possible compositional changes during the transformation rather than to determine precise local stoichiometries.

For microstructural analysis of the post‐annealed specimens, the dried crystals were deposited on carbon tape, and backscattered electron (BSE) imaging was performed using a field‐emission Zeiss GeminiSEM 560 scanning electron microscope operated at an accelerating voltage of 7 kV.

In situ heating STEM experiments were carried out using a probe‐ and image‐aberration‐corrected Thermo Fisher Scientific Spectra 300 S/TEM equipped with an X‐FEG source, operated at an accelerating voltage of 300 kV. A Protochips Fusion Select heating holder was employed. Samples were prepared by dispersing a few mg of the 24 h crystals in 2 mL of toluene. Next, a drop of 3 µl of the diluted solution was cast onto a Fusion Thermal E‐chip, with a central silicon nitride membrane supported by a silicon substrate, incorporating a 3 × 3 array of nine electron‐transparent holes (8 µm diameter, 12 µm spacing), and a carbon support film spanning the holes providing mechanical stability for the deposited microcrystals. Temperature control during the in situ experiment was achieved using the Clarity Fusion Select software, and the E‐chip was calibrated prior to the experiment using the manufacturer‐provided calibration parameters associated with the specific chip box ID.

Time‐resolved STEM‐HAADF imaging was continuously recorded throughout the heating and cooling cycles, enabling direct observation of the temperature‐dependent morphological evolution of individual microcrystals.

^1^H Nuclear Magnetic Resonance (NMR) Spectroscopy. All the ^1^H NMR spectra were acquired at 298K on a Bruker Avance III 600 MHz spectrometer equipped with a 5 mm QCI cryoprobe with a z‐shielded pulsed‐field gradient coil. 5‐mm disposable tubes (Bruker) filled with 500 µL of the sample solution prepared previously by dissolving 30 mg of crystals in 600 µL of the DMSO‐d6, were used for NMR analysis. Before the acquisition, the automatic matching and tuning, homogeneity adjustment, and 90° pulse optimization were performed [49]. 16 transients were accumulated at a fixed receiver gain (7.12), with 65536 complex data points and an inter‐pulse delay of 30 s, over a spectral width of 20.83 ppm, with the offset set to 6.18 ppm. The benzyl ammonium concentration was measured by comparing the integrated peaks intensity (i.e. aromatic proton from 7.3 to 7.5 ppm) to that generated by an external standard 15 mm solution of DMS (DiMethylSulfon, TraceCERT, 99.99%) in DMSO‐d6, normalizing, each peak to the number of resonances (H) generating the signal, by means of the PULCON (PUlse Length Based Concentration Determination) procedure [50].

### 4.5 Thermal Analysis

Thermogravimetry analysis (TGA) of the dried 24h‐shaken (BZA)_2_PbBr_4_ powder was performed using a TGA Q500 system, from room temperature to 800°C, with a ramping rate of 5°C/min in a nitrogen atmosphere under a flow rate of 50 mL/min.

To investigate possible phase transformations as a function of temperature, differential scanning calorimetry (DSC) measurements were performed using a Discovery DSC 250 instrument equipped with a refrigeration system (Waters‐TA Instruments, Discovery Refrigerating Cooling System RCS90) under a continuous nitrogen flow of 50 mL min^−^^1^. Approximately 5 mg of the 24 h shaken sample was sealed in an aluminum (not hermetic TZero) pan. The DSC protocol consisted of an initial equilibration at −90°C, followed by a first heating cycle from −90°C to 130°C at 5°C min^−^^1^, with a 1 min isothermal hold at the upper temperature. A first cooling cycle from 130°C to −90°C at the same rate was then carried out, followed by a 1 min isothermal segment. The program was subsequently repeated for a second heating cycle (from −90°C to 130°C) and a second cooling cycle (from 130°C to −90°C), each performed at 5°C min^−^^1^ with identical isothermal holds. All DSC scans were acquired under identical atmospheric and ramping conditions.

### 4.6 Optical Characterization

Ex situ PL measurements were performed on a Tecan Spark multimode microplate reader. The samples were placed in a 96‐well quartz plate, and PL spectra were collected in the bottom‐detection configuration with excitation at 360 nm. For in situ PL measurements, an Avantes AvaSpec‐USL, coupled to an optical fiber Y‐bundle, was used. The probe tip of the optical fiber was placed near the reaction flask under agitation, and PL spectra were collected using a UV LED (λexc = 365 nm). PL spectra were collected every 15 min over a total monitoring time of 25 h.

## Author Contributions

L.G.B. and S.K. contributed equally to this work, and M.P.A. designed and supervised the research. L.G.B. prepared all the samples, performed the structural characterization of the time‐driven evolution, and performed the in situ PL and thermal experiments. S.K. performed all the imaging analysis and conducted the in situ XRD and TEM studies, along with the DSC and TGA analysis. B.D. and Q.E. assisted with the structural and optical analysis, respectively. L.G. and S.M. performed the NMR and XRD analysis, respectively. G.D. supervised the in situ and ex situ TEM studies. All authors discussed the progress of this study and reviewed the manuscript.

## Conflicts of Interest

The authors declare no conflicts of interest.

## Supporting information




**Supporting File 1**: advs76733‐sup‐0001‐SuppMat.pdf.

**Supporting File 2**: advs76733‐sup‐0002‐MovieS1.mp4.

**Supporting File 3**: advs76733‐sup‐0003‐MovieS2.mp4.

## Data Availability

The data that support the findings of this study will be published on the IIT Dataverse and Zenodo and linked to a DOI.
