# Supplementary material for: Programming Structural Symmetry and Emission in Low‐Dimensional Hybrid Perovskites via Time and Temperature
Source: Adv Sci (Weinh). 2026 Jul 23:e76733. Online ahead of print. doi: 10.1002/advs.76733 (PMC13393262; doi:10.1002/advs.76733)
Supplement: Supplementary file 1 — Supporting File 1: advs76733‐sup‐0001‐SuppMat.pdf. [file ADVS-9999-e76733-s001.pdf]

Supporting Information for

Programming Structural Symmetry and Emission in Low-Dimensional Hybrid Perovskites via Time and Temperature

Luiz G. Bonato[†ŧ], Sirous Khabbaz Abkenar[†¥ŧ], Balaji Dhanabalan[†], Quentin Evrard[†], Luca Goldoni[§], Sergio Marras[§], Giorgio Divitini[¥], and Milena P. Arciniegas[†]*

†Automated Nanomaterials Engineering, [¥]Electron Spectroscopy and Nanoscopy, and [§]Materials Characterization Facility. Center for Convergent Technologies, Istituto Italiano di Tecnologia, Via Morego 30, 16163, Genova, Italy.

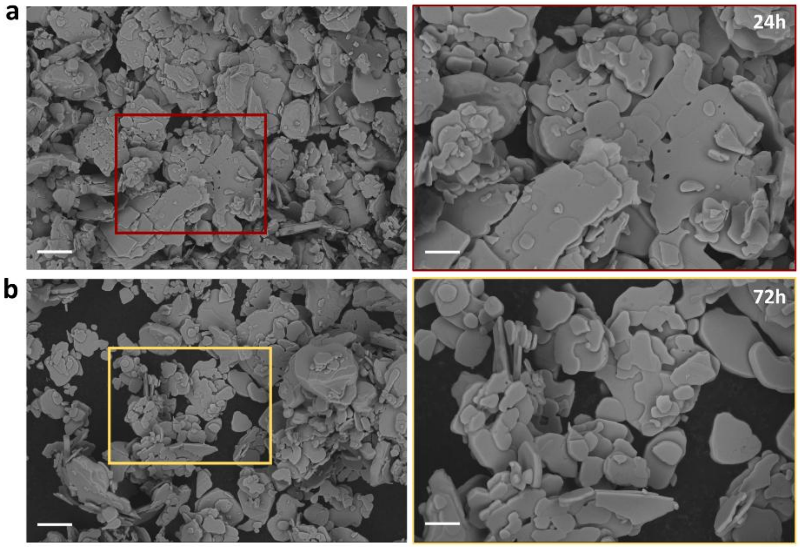

Figure S1. SEM images of the samples prepared without shaking at 24h (a) and 72h (b), collected with different magnifications, showing that the structures preserve their platelet-like morphology. Scale bars: 5 µm (low magnification, left) and 2 µm (high magnification, right).

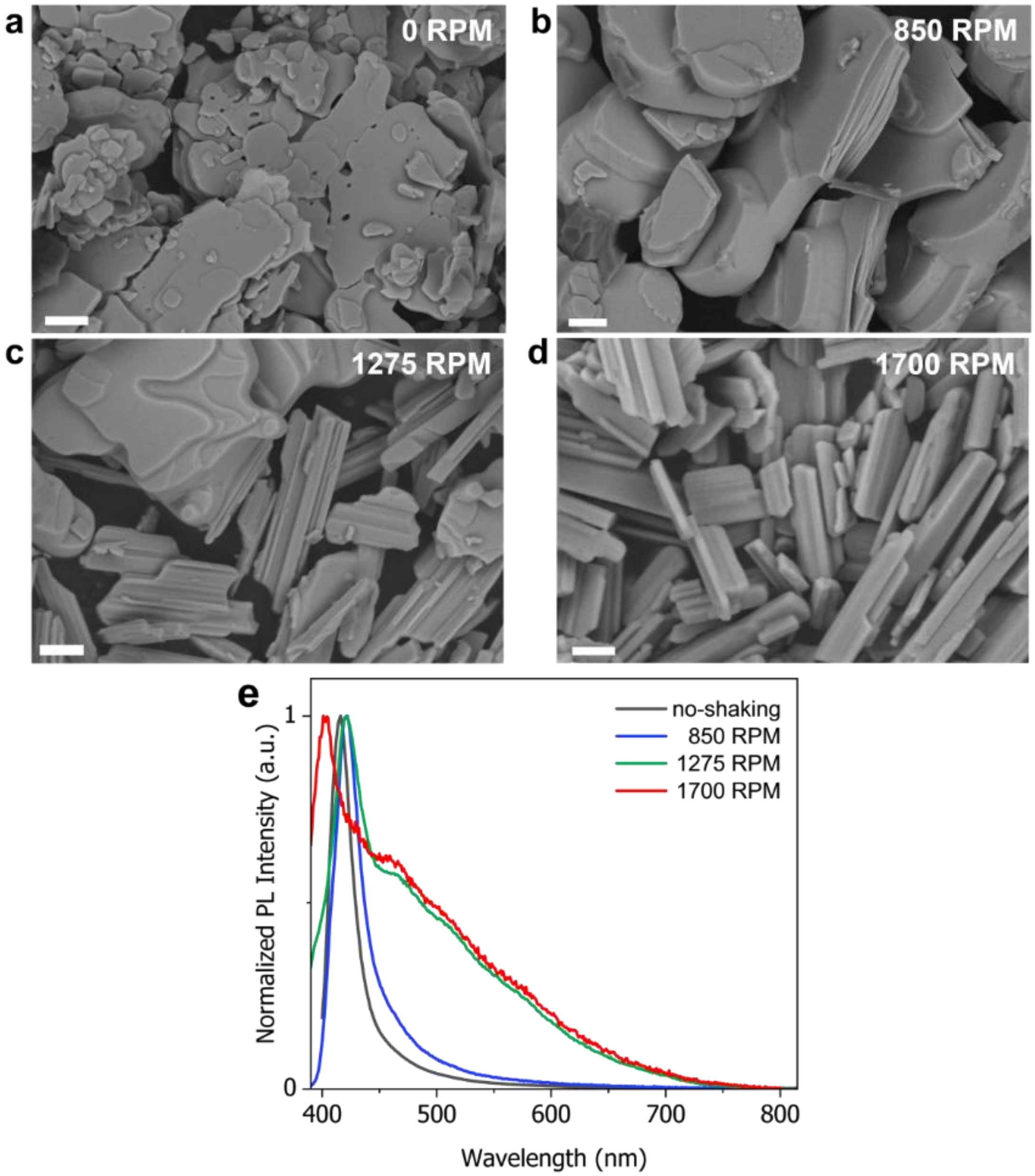

Figure S2. SEM images collected from the samples prepared under different rpm shaking condition for 24 h: (a) 0 rpm; (b) 850 rpm; and (c) 1700 rpm. Scale bars: 2 µm. (d) Normalized PL spectra of the corresponding samples acquired under 365 nm excitation.

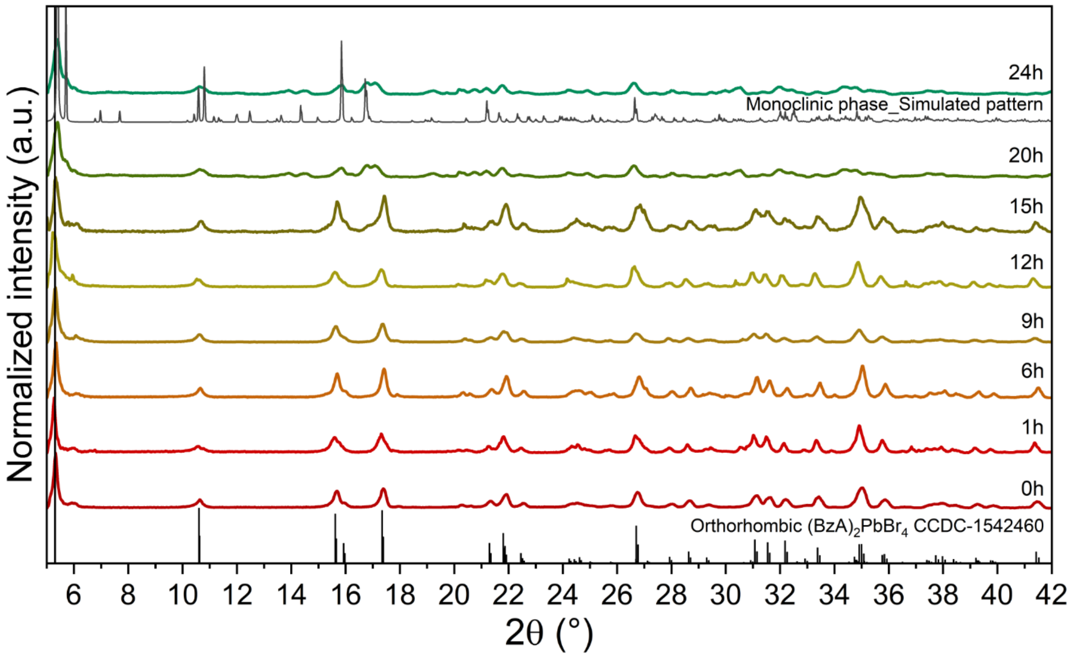

Figure S3. Full XRD patterns collected from the crystal after different shaking times. The pattern from the reference $(BzA)_2PbBr_4$ orthorhombic structure is displayed in vertical lines. As a reference, the simulated pattern extracted from our 3D electron data presented in Ref. 1 is shown in black.

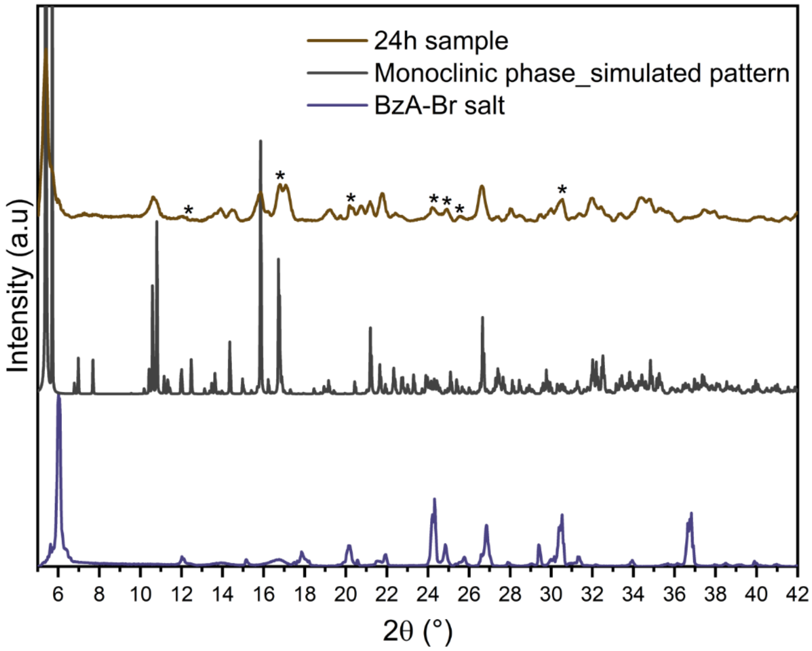

Figure S4. XRD pattern collected from the crystals after 24 h of shaking (in brown) and the benzylammonium-bromide (BzA-Br) salt (in violet). The asterisk highlights the reflections associated with the salt in the 24 h samples, indicating the presence of traces in the ensemble of crystals used for the analysis. As a reference, the simulated pattern extracted from our 3D electron data presented in Ref. 1 is shown in black.

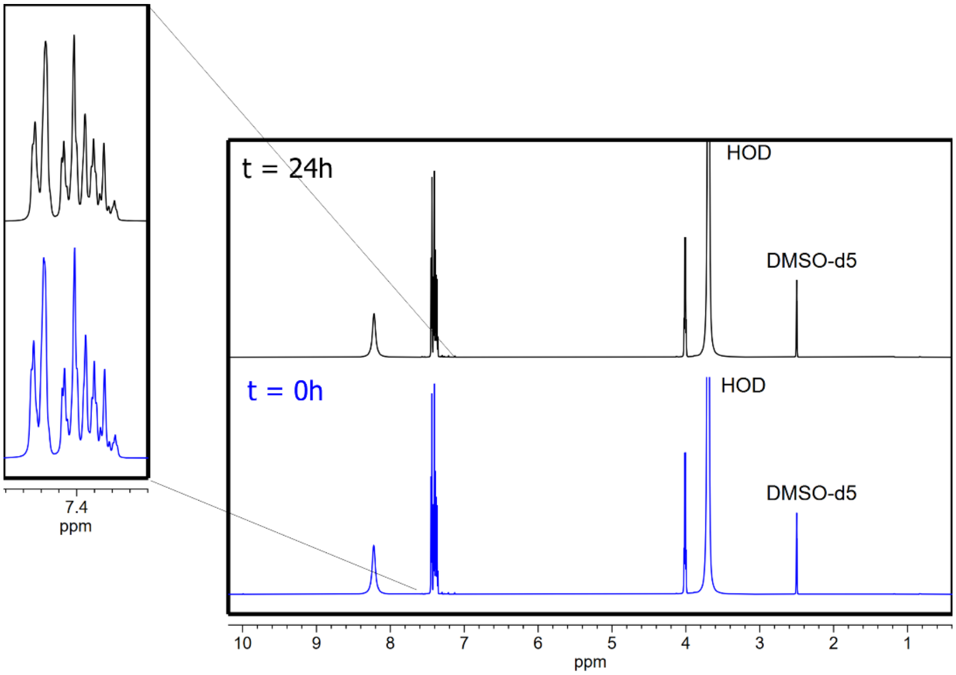

Figure S5. Quantitative 1H-NMR spectra of samples collected at 0 h and 24 h after dissolution in DMSO-d6, used to determine the concentration of benzylammonium.

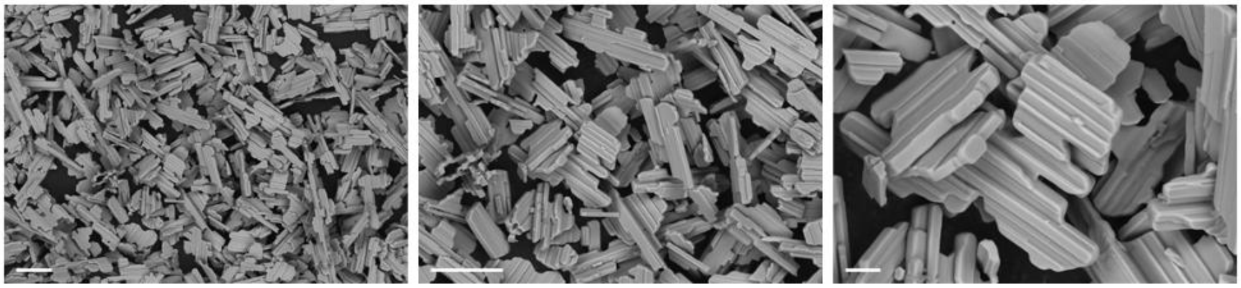

Figure S6. SEM images of the crystals collected after extended shaking time (72 h), showing the stabilized elongated microcrystal morphology with faceted edges and longitudinal grooves. Images are shown at different magnifications. No further morphological evolution is observed after 24 h. Scale bars: 10 µm (left, middle), and 2 µm (right).

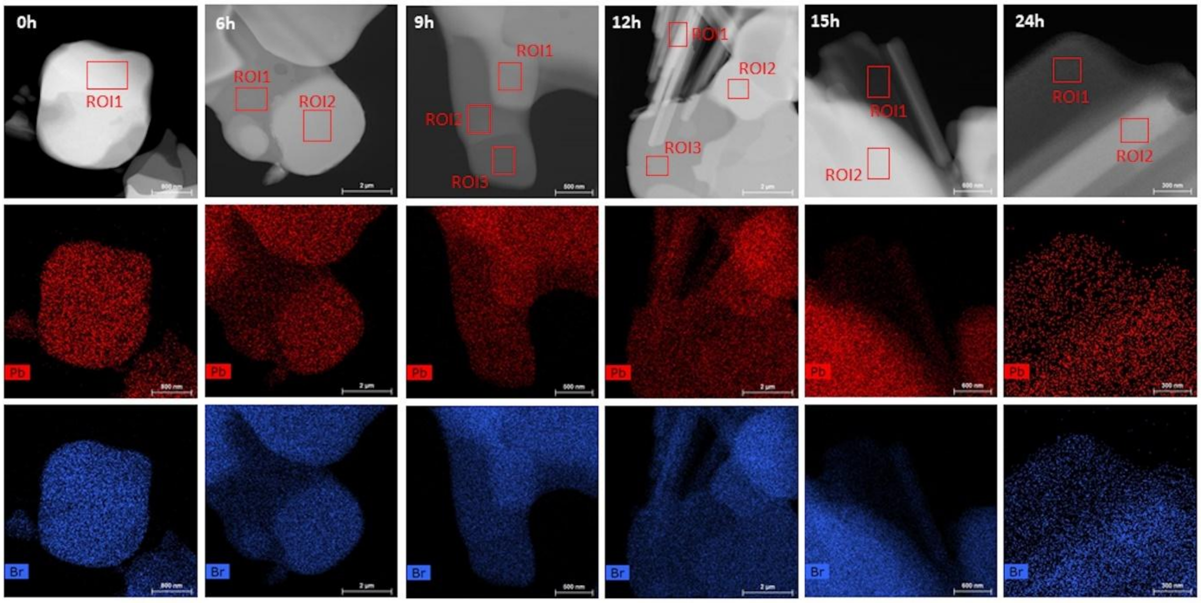

Figure S7. STEM-HAADF images with corresponding STEM-EDX elemental maps Pb (red) and Br (blue) acquired from representative crystals collected at selected shaking times. Regions of interest (ROIs) marked on the HAADF images were selected for local composition analysis and integrated to obtain the quantitative values summarized in Table S1. The images are presented to identify representative regions with different contrast features and assess possible compositional variations throughout the transformation; differences in magnification reflect crystal size and imaging conditions and are not intended for quantitative morphological comparison.

Table S1. Quantitative STEM-EDX compositional analysis of representative samples extracted at selected shaking times. Atomic percentages of Pb and Br were obtained from integrations over the regions of interest (ROIs) indicated in the corresponding STEM-HAADF images in Figure S7. The number of analyzed ROIs varies between samples according to crystal availability and data quality. Variations between individual ROIs may arise from differences in local crystal thickness, crystal orientation, and detector geometry, and should therefore be interpreted qualitatively. The analysis is intended to evaluate possible compositional changes associated with the structural transformation and does not reveal evidence of systematic compositional segregation.

Specimen	ROI	Pb (at. %)	Br (at. %)	Br:Pb
0h	#1	20.4	79.6	3.9
6h	#1	19.4	80.6	4.1
	#2	19.3	80.7	4.2
9h	#1	19.9	80.1	4.0
	#2	20.4	79.6	3.9
	#3	19.6	80.4	4.1
12h	#1	17.2	82.8	4.8
	#2	18.5	81.5	4.4
	#3	20.2	79.8	3.9
15h	#1	18.4	81.6	4.4
	#2	19.0	81.0	4.2
24h	#1	24.5	75.5	3.0
	#2	19.3	80.7	4.1

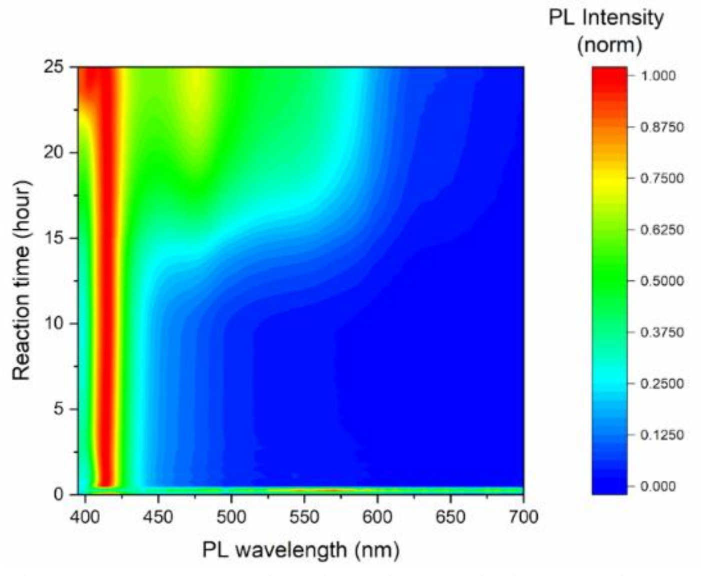

Figure S8. PL intensity contour map showing the evolution of the emission profile during continuous shaking, highlighting the emergence and growth of the broadband component after ~12 h.

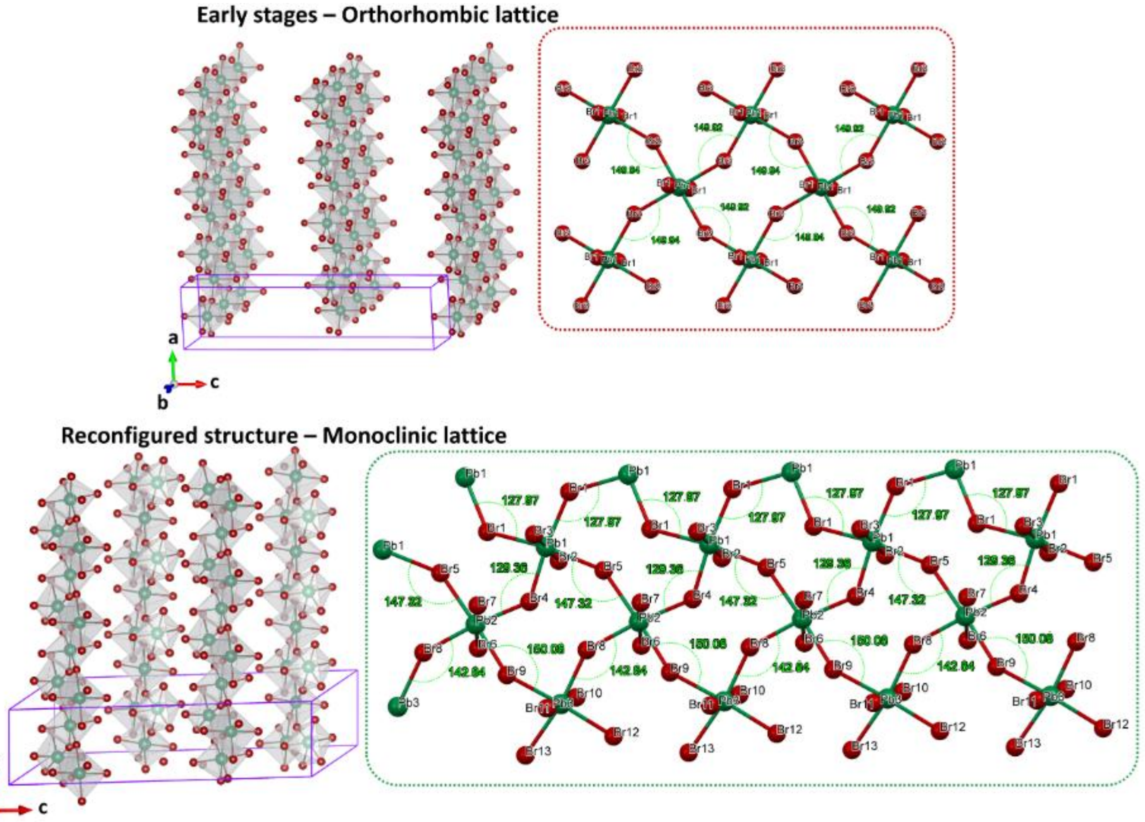

Figure S9. Crystallographic views from the BzA_2PbBr_4 crystals observed at the early stage of the transformation (from 0 h to 9h; CCDC 1542460) and the final stage (24 h),[1] showing the reconfigured inorganic lattice with a zig-zag conformation and the corresponding angles listed in Table 1 of the main document.

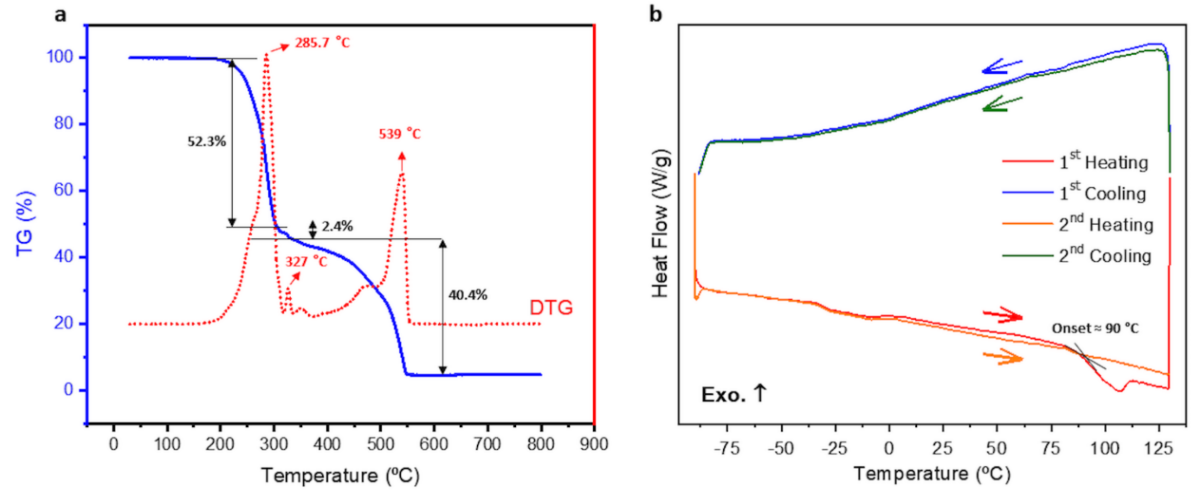

Figure S10. Thermal analysis of the crystals obtained after 24 h of agitation. **a,** Thermogravimetric (TG) and derivative thermogravimetric (DTG) curves showing the mass loss profile as a function of temperature. **b,** Differential scanning calorimetry (DSC) thermograms recorded between –90 °C and 130 °C at a heating/cooling rate of 5 °C min^{-1}, including the 1st heating (red), 1st cooling (blue), 2nd heating (orange), and 2nd cooling (green) cycles. A distinct endothermic event with an

onset at ~90 °C is observed only during the first heating cycle, indicating a one-time solid-solid phase transition. Heat flow is plotted with exothermic processes in the upward direction.

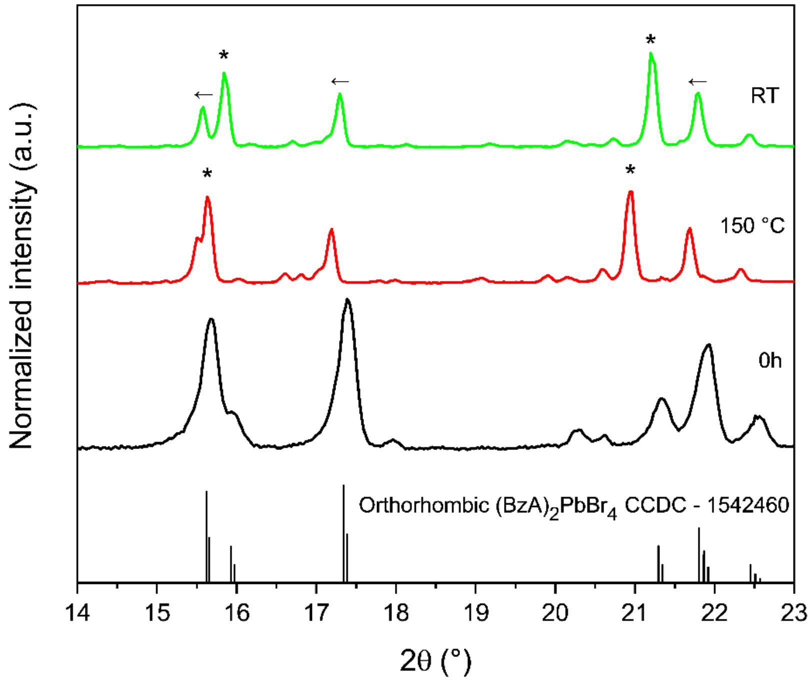

Figure S11. Comparison of powder X-ray diffraction (XRD) patterns of the 0 h $(BzA)_2PbBr_4$ orthorhombic phase and the 24 h sample after thermal treatment at 150 °C, followed by cooling to room temperature.

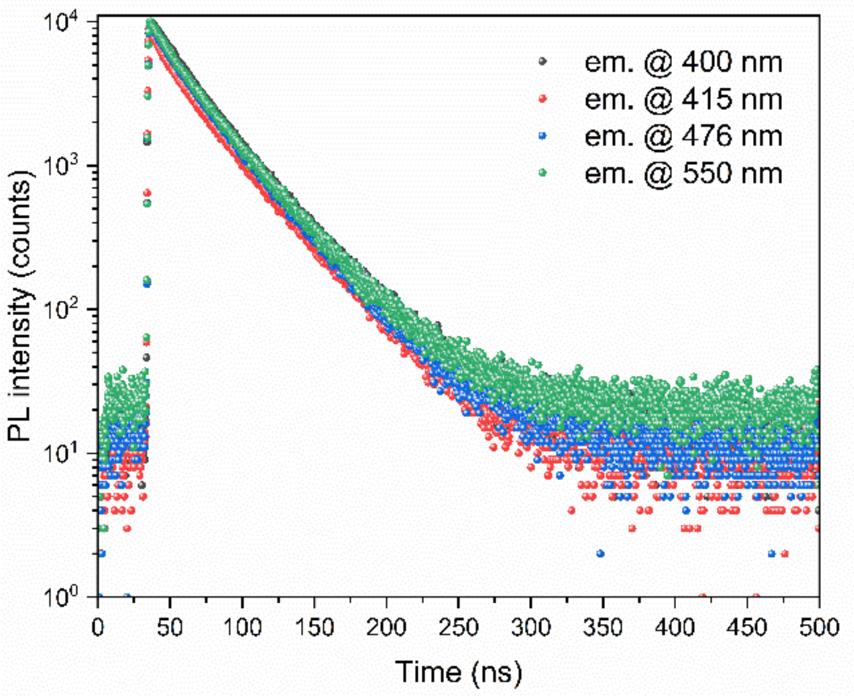

Figure S12. PL decay curves acquired from the 24 h samples at 400 nm, 415, nm, 476 nm and 550 nm emission peaks showing similar decay profiles.

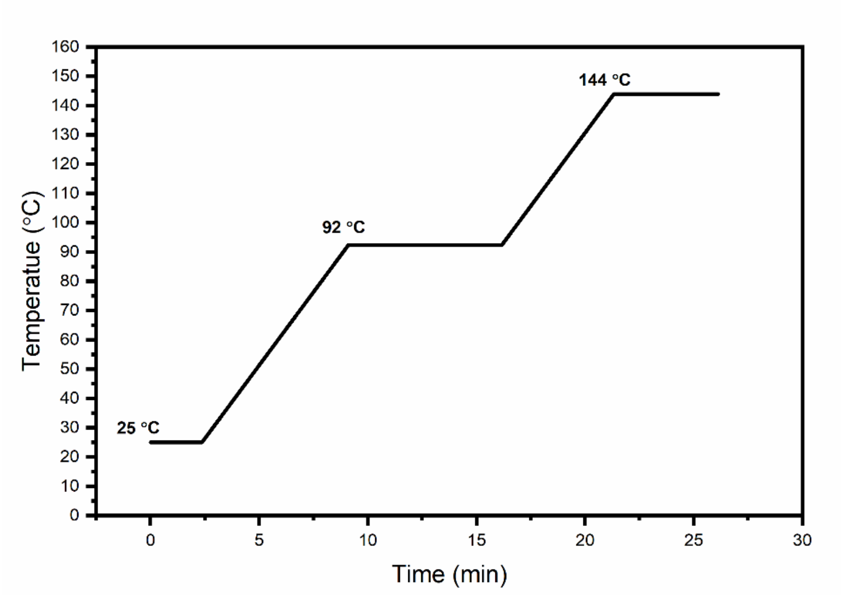

Figure S13. Temperature profile used for in situ heating STEM showing stepwise heating from 25 °C to ~145 °C with an intermediate isothermal hold at ~92 °C prior to further ramping and stabilization at ~145 °C.

References

[1] A. Prabhakaran, Q. Evrard, D. Marchetti, S. Khabbaz Abkenar, L. G. Bonato, B. Dhanabalan, I. Andrusenko, S. Lauciello, P. Solokha, S. De Negri, L. Goldoni, M. D. Faye Diouf, L. Manna, G. Divitini, M. Gemmi, M. P. Arciniegas, Small Structures 2025, 6.
